# Parents’ Awareness and Attitude Toward Pediatrics Eye Diseases in Makkah, Saudi Arabia: A Cross-Sectional Study

**DOI:** 10.7759/cureus.38366

**Published:** 2023-05-01

**Authors:** Abdulaziz H Almogbel, Nasser Al Shanbari, Ibrahim S Alibrahim, Shajn S Alsaadi, Hajar S Algarni, Abdullah S Alshanbari, Reda Goweda

**Affiliations:** 1 Department of Medicine and Surgery, College of Medicine, Umm Al-Qura University, Makkah, SAU; 2 Department of Community Medicine, Umm Al-Qura University, Makkah, SAU; 3 Department of Family Medicine, Faculty of Medicine, Suez Canal University, Ismailia, EGY

**Keywords:** saudi arabia, parents, eye disease, children, awareness

## Abstract

Background: The necessity of early detection and parents' knowledge of pediatric eye conditions and eye care is crucial, not only because of the severe complications it can carry, like blindness, but also due to the availability of preventive measures and the importance of vision in a child's growth and social life. Therefore, this study aimed to evaluate the level of awareness of and attitudes toward children's eye diseases of parents in Makkah City, Saudi Arabia.

Methods: This descriptive, cross-sectional questionnaire-based study was conducted in Makkah, Saudi Arabia, from September to December 2022. A self-administered questionnaire was randomly distributed to fathers and mothers of children younger than 18 through social media platforms.

Results: A total of 470 parents who met the study’s inclusion criteria completed the study questionnaire. The results showed that 72.8% of the parents had poor awareness of pediatric eye diseases, 24.5% had good awareness, and 2.8% had excellent awareness. With regard to the symptoms that might prompt parents to take their children to an eye specialist, eye redness had the highest percentage (24.5%). It was also shown that 68.5% would allow their children to undergo eye surgery, if necessary, while most of those who refused to do so (11.3%) cited fear of the surgical outcome as the cause of their refusal.

Conclusion: Our study findings show inadequate parental knowledge about common pediatric eye diseases and eye care practices for children. Therefore, awareness and education programs targeting both parents are recommended to raise the parents’ level of awareness of pediatric eye diseases and to improve their attitudes toward the same.

## Introduction

The Convention on the Rights of the Child defines a child as “every human being below the age of eighteen years” [1]. Saudi Arabia’s population in 2021 was 34,110,821, with children under 19 years accounting for approximately 10,781,451 (31.6%) of the total [2]. Given that vision is our primary contact with the outside world, vision difficulties may significantly negatively impact a child’s development [3]. They may also negatively affect a child’s social life, motor skills, education, and other fundamental life skills [3]. The visual system develops before birth and continues until after delivery [4]. The sense of sight develops swiftly within the child’s first year and becomes the primary sense in the sixth month [4]. During this developmental period and until the child is six years old, the eyes are susceptible to various disorders that may impair vision [4]. In Jazan, Saudi Arabia, the most prevalent pediatric eye diseases were strabismus, refractive error, ocular trauma, and conjunctival and corneal infections (36.1%, 26.5%, 7.5%, and 7.3%, respectively) [5]. In Dammam, Saudi Arabia, the most significant pediatric eye diseases were refractive error and strabismus (44.4% and 38%, respectively) [6]. In the last study conducted in Madinah, Saudi Arabia, in February 2022, about parents’ awareness of pediatric eye diseases, 40.5% of the parents mentioned that their children had an eye disease [7].

Each year, 500,000 children worldwide experience blindness in the Middle East; childhood blindness accounts for 4.1% of all blindness cases [8,9]. According to the World Health Organization, blindness can be prevented in 75% of eye disease cases, regardless of age, through therapeutic and preventive measures [10]. Early detection and parents’ knowledge of pediatric eye conditions and proper eye care are necessary not only because such conditions may result in blindness that can be prevented but also due to the importance of vision in a child’s growth and social life [8]. However, limited research has focused on parents’ awareness of and attitudes toward pediatric eye conditions and vision loss in Saudi Arabia [11,12]. Studies have been performed in Riyadh, Arar, and Madinah. They have concluded that parents' awareness and knowledge of pediatric eye diseases in such cities are unsatisfactory and that more education programs are needed [6-8]. Therefore, we conducted this study to evaluate parents' awareness and attitudes toward pediatric eye diseases in Makkah City, Saudi Arabia.

## Materials and methods

Study design and ethical consideration

A descriptive, cross-sectional, questionnaire-based study was conducted involving parents of children younger than 18 years in Makkah City from September to December 2022 after obtaining ethical approval for the study from the Umm Al-Qura University research ethics committee (approval number HAPO-02-K-012-2022-11-1246).

Study setting and participants

Both the fathers and mothers of children younger than 18 years were asked to participate in the present study. Those who refused to participate in the study, resided outside Makkah City, or worked in an eye care-related medical field were excluded from the study.

Sample size

The General Authority for Statistics in Saudi Arabia estimates that 1,908,000 people lived in Makkah City in 2017. Using convenience sampling methods and the OpenEpi software, we calculated the sample size and found that to obtain a 95% confidence level and a 5% acceptable error margin covering the Makkah City population, 384 parents had to participate in the study [13].

Study procedure and questionnaire design

The self-administered questionnaire used in the present study was adapted from a previously published study [7]. It was translated into Arabic by two independent bilingual translators, then a team of experts and translators evaluated the translation to resolve any issues. A reverse translation from Arabic to English was also conducted to ensure that no issues would affect the research tool. Before data collection, 10 respondents were given a copy of the questionnaire. The medical terms were simplified based on the feedback obtained, and some questionnaire items were reconstructed. The final questionnaire consisted of four parts and had 31 closed-ended questions. The first part comprised demographic questions about gender, age, education, occupation, income, and nationality. The second part contained five questions that evaluated how much the parents knew about eye care. The third section contained 11 questions about eye diseases in children. Finally, the fourth section contained six questions about eye care practices. The questionnaire was sent to the study participants through online social media networks like Facebook, Twitter, Instagram, Snapchat, and WhatsApp.

Statistical analysis

The data were collected, reviewed, and then fed to Statistical Package for Social Sciences (SPSS) version 21 (IBM Corp., Armonk, NY). All statistical methods used were two-tailed with an alpha level of 0.05, considering significance if the p-value is less than or equal to 0.05. Regarding knowledge, each correct answer was given a one-point score. Overall knowledge level regarding pediatric eye diseases was assessed by summing up discrete scores for different correct knowledge items. If the total score was 75% or more of the total possible score, the level of knowledge was considered to be excellent. Scores between 50% and 74% were considered good, and scores less than 50% were considered poor. The categories of good and excellent knowledge were combined to make the category acceptable. Descriptive analysis was done by prescribing frequency distribution and percentage for study variables, including parents' personal data and children's eye problems. In addition, participants' knowledge and practice toward pediatric eye diseases were tabulated while overall knowledge level was graphed. Cross tabulation was used for showing the distribution of parents' overall knowledge level by their personal data and other factors and for assessing the relation between parents' knowledge and their practice using the Pearson chi-square test for significance and exact probability test, if there were small frequency distributions.

## Results

A total of 470 parents fulfilling the inclusion criteria completed the study questionnaire. Parents' ages ranged from 18 to 65 years, with a mean age of 46.2 ± 13.9 years old. The exact of 284 (60.4%) parents were males, and 451 (96%) were Saudis. As for educational level, 301 (64%) were university graduates, and 135 (28.7%) had below university level of education. A total of 241 (51.3%) were employed, and 183 (38.9%) were not employed. Monthly income was sufficient among 236 (50.2%) parents and insufficient among 40 (8.5%) (Table 1).

**Table 1:** Personal data of the parents in Makkah, Saudi Arabia

Personal data	No.	%
Age in years		
18-40	188	40.0%
41-60	270	57.4%
>60	12	2.6%
Gender		
Male	186	39.6%
Female	284	60.4%
Nationality		
Saudi	451	96.0%
Non-Saudi	19	4.0%
Educational level		
Read and write	34	7.2%
Below university	135	28.7%
University / above	301	64.0%
Employment		
Unemployed	183	38.9%
Employed	241	51.3%
Retired	46	9.8%
Monthly income		
Insufficient	40	8.5%
Intermediate	194	41.3%
Sufficient	236	50.2%

Exact 121 (25.7%) reported that they had a child with refractive error, 32 (6.8%) had a child with amblyopia, and 252 (53.6%) had a child with no eye diseases. Exactly 177 (92.7%) of children with eye disorders go to school. Among others who did not attend school, 24 (23.8%) reported it was because the parents did not know where to enroll their children, 22 (21.8%) of their parents think that the child cannot learn without seeing well, while six (5.9%) were afraid that the child would not cope. As for the preferred option for disseminating information about children's visual problems, the most reported were social media (31.3%), physicians (29.8%), health education campaigns (28.7%), and the internet (4.7%) (Table 2).

**Table 2:** Children's eye diseases reported by parents in Makkah, Saudi Arabia HE: Health education.

Eye problems	No	%
Child eye problems		
None	252	53.6%
Refractive error	121	25.7%
Others	52	11.1%
Amblyopia	32	6.8%
Congenital glaucoma	8	1.7%
Congenital cataract	5	1.1%
If your child has a vision problem, does he/she go to school?		
Yes	177	92.7%
No	14	7.3%
What prevents your child who has a vision problem from going to school?		
Afraid that the child will not cope	6	5.9%
The child will be teased by his peers	5	5.0%
He cannot learn without seeing well	22	21.8%
I don't know to put him in any school	24	23.8%
Others	44	43.6%
What is the preferred option for disseminating information about children's visual problems?		
Social media	147	31.3%
Physician	140	29.8%
HE campaigns	135	28.7%
Internet	22	4.7%
Posters	16	3.4%
Mass media	10	2.1%

A total of 38.7% know that amblyopia is poor vision in one or both eyes. Regarding causes of amblyopia, most of the parents perceive the causes of amblyopia are smart devices/TV (17.2%), hereditary causes (11.5%), and squint (7.9%), while 50.9% do not know about the causes. An exact 23.6% said that covering the healthy eye is a treatment method for amblyopia, 16.2% know about wearing glasses, and 10.4% know about eye muscle exercises. A total of 63.6% reported that early treatment leads to better results. Considering the consequences of amblyopia, the most reported were decreased visual acuity (36.6%), while 45.5% do not know about it. As for cataract, only 16.6% of the parents know that it is opacity at the eye lens, but 44.9% think that cataract affects children, while 19.8% said that cataract causes permanent blindness. Considering glaucoma, only 12.3% think that it increases eye pressure, and 25.5% think that it affects children, while 22.6% reported that congenital glaucoma leads to blindness (Table 3).

**Table 3:** Parents' awareness regarding pediatric eye diseases in Makkah, Saudi Arabia

Eye disease	Items	(Correct answer)	
		No.	%
Amblyopia	What is amblyopia?		
	Poor vision in one or both eyes	182	38.7%
	What are the causes of amblyopia?		
	Refractive errors	33	7.0%
	Treatment of amblyopia		
	Cover normal eye	111	23.6%
	Will early treatment lead to better results?		
	Yes	299	63.6%
	What are the consequences of amblyopia?		
	Decreased visual acuity	172	36.6%
Cataract	What is a cataract?		
	Opacity at eye lens	78	16.6%
	Do cataracts affect children?		
	Yes	211	44.9%
	Do cataracts cause permanent blindness?		
	No	38	8.1%
Glaucoma	What is glaucoma?		
	Damage to the optic nerve due to high pressure	72	15.3%
	Can glaucoma affect children?		
	Yes	120	25.5%
	Can congenital glaucoma lead to blindness?		
	Yes	106	22.6%

A total of 65.6% of the parents reported that their children had an eye or vision exam at the age of 5-10 years, while 24% of them did not. Routine eye examination every year was reported by 37.7% of the study parents, while 21.7% did when the child had an eye problem. On asking about symptoms that might prompt parents to take their child to an eye specialist, eye redness was reported among 24.5% of the parents, followed by frequent rubbing of the eye (18.9%), double vision complaint (16.8%), playing with toys from a close distance (16%), and staring eyes (11.1%). A total of 81.1% of the parents accept the child wearing glasses. Also, 68.5% allowed their children to undergo eye surgery if necessary. Among refusers, the most reported cause was fear of the outcome (11.3%), being not sure of surgery (8.9%), and the operation cost (7.4%) (Table 4).

**Table 4:** Parents' practice and attitude toward pediatric eye diseases in Makkah, Saudi Arabia

Items	No	%
Has your child ever had any kind of eye or vision exam? And at what age?		
Less than 1 year	28	6.0%
1-5 years	99	21.1%
5-10 years	113	24.0%
10-15 years	73	15.5%
Never	157	33.4%
How often should a child undergo a routine eye examination?		
Every year	177	37.7%
Every 2 years	68	14.5%
Every 5 years	33	7.0%
When the child has a problem	102	21.7%
I don't know	90	19.1%
What are the symptoms that might prompt you to take your child to an eye specialist?		
Eye redness	115	24.5%
Staring eyes	52	11.1%
Double vision complaints	79	16.8%
Playing with toys from a close distance	75	16.0%
Frequent rubbing of the eye	89	18.9%
Tilting the head to one side	41	8.7%
Excessive tear production	19	4.0%
Do you accept the child wearing glasses?		
Yes	381	81.1%
No	89	18.9%
Would you allow your child to undergo eye surgery if necessary?		
Yes	322	68.5%
No, the operation cost	35	7.4%
No, fear of the outcome	53	11.3%
No, it would cause more damage to the eyes	11	2.3%
No, I'm not sure	42	8.9%
No, cultural and social barriers	7	1.5%

**Figure 1:**
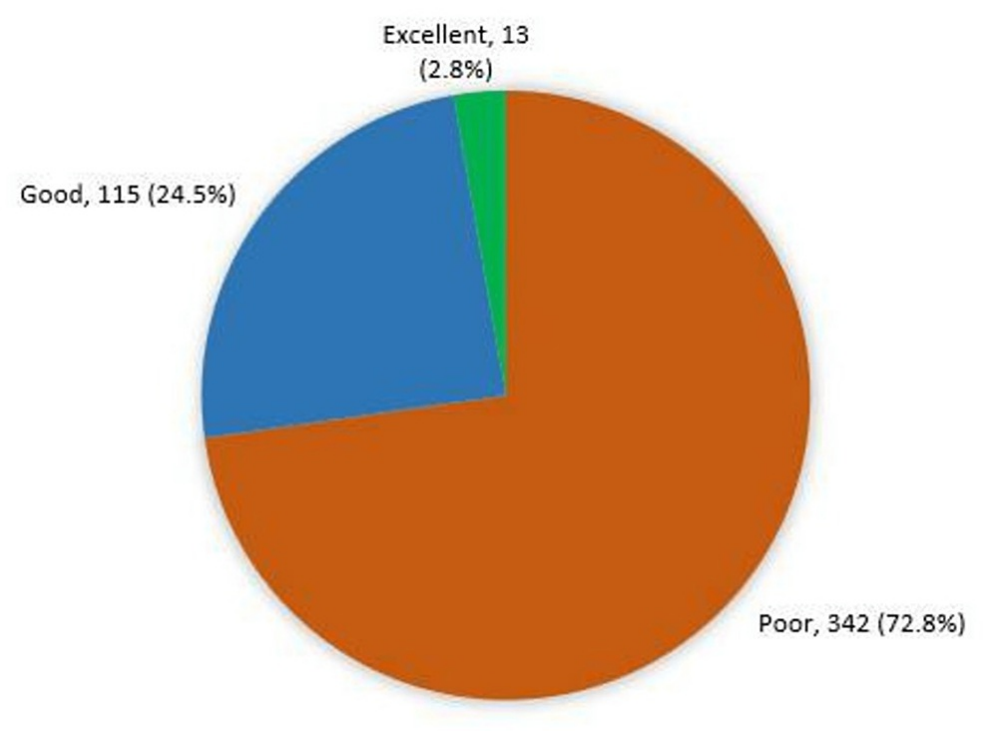
Overall parents' awareness level of pediatric eye diseases in Makkah, Saudi Arabia

A total of 72.8% of the parents had a poor awareness level regarding pediatric eye diseases, 24.5% had a good awareness level, and 2.8% had excellent awareness (Figure 1).

**Figure 2:**
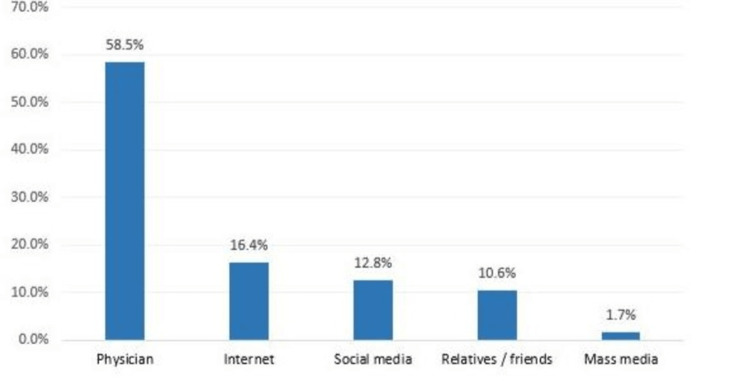
Source of the parent's information regarding pediatric eye diseases in Makkah, Saudi Arabia

Regarding the source of parents' information for pediatric eye diseases, the most reported were physicians (58.5%), internet (16.4%), social media (12.8%), and relatives/friends (10.6%) (Figure 2).

Exact 33% of parents aged 18-40 years had good/excellent awareness of pediatric eye diseases versus 8.3% of parents older than 60 years with recorded statistical significance (p = .036). Also, 28.2% of Saudi parents had good/excellent awareness compared to 5.3% of non-Saudis (p = .028). In addition, good/excellent awareness was reported among 32.4% of employed parents compared to 20.8% of the unemployed group (p = .029) (Table 5).

**Table 5:** Factors associated with parents' awareness regarding pediatric eye diseases in Makkah, Saudi Arabia P: Pearson's X^2^ test; $: Exact probability test; * P < 0.05 (significant).

Factors	Overall knowledge level				p-values
	Poor		Good/Excellent		
	No	%	No	%	
Age in years					.036*^$^
18-40	126	67.0%	62	33.0%	
41-60	205	75.9%	65	24.1%	
>60	11	91.7%	1	8.3%	
Gender					.619
Male	133	71.5%	53	28.5%	
Female	209	73.6%	75	26.4%	
Nationality					.028*^$^
Saudi	324	71.8%	127	28.2%	
Non-Saudi	18	94.7%	1	5.3%	
Educational level					.206
Read and write	24	70.6%	10	29.4%	
Below university	106	78.5%	29	21.5%	
University/above	212	70.4%	89	29.6%	
Employment					.029*
Unemployed	145	79.2%	38	20.8%	
Employed	163	67.6%	78	32.4%	
Retired	34	73.9%	12	26.1%	
Child eye problems					.556
None	181	71.8%	71	28.2%	
Refractive error	88	72.7%	33	27.3%	
Congenital cataract	3	60.0%	2	40.0%	
Congenital glaucoma	6	75.0%	2	25.0%	
Amblyopia	21	65.6%	11	34.4%	
Others	43	82.7%	9	17.3%	
What is the source of your information about eye diseases?					.491
Physician	196	71.3%	79	28.7%	
Social media	47	78.3%	13	21.7%	
Internet	53	68.8%	24	31.2%	
Relatives/friends	39	78.0%	11	22.0%	
Mass media	7	87.5%	1	12.5%	

A total of 29.7% of those with good/excellent awareness never assessed their child's vision (examination) versus 34.8% of others with poor awareness levels (p = .048). Also, 50% of parents with good/excellent knowledge know that their child should undergo a routine eye examination annually versus 33% of others with poor awareness levels (p = .001) (Table 6).

**Table 6:** Relationship between the parents' awareness and their practice regarding pediatric eye diseases in Makkah, Saudi Arabia P: Pearson's X^2^ test; $: Exact probability test; * P < 0.05 (significant).

Practice	Overall knowledge level				p-values
	Poor		Good/Excellent		
	No	%	No	%	
Has your child ever had any kind of eye or vision exam? And at what age?					.048*
Less than 1 year	16	4.7%	12	9.4%	
1-5 years	70	20.5%	29	22.7%	
5-10 years	89	26.0%	24	18.8%	
10-15 years	48	14.0%	25	19.5%	
Never	119	34.8%	38	29.7%	
How often should a child undergo a routine eye examination?					.001*^$^
Every year	113	33.0%	64	50.0%	
Every 2 years	53	15.5%	15	11.7%	
Every 5 years	20	5.8%	13	10.2%	
When the child has a problem	78	22.8%	24	18.8%	
I don't know	78	22.8%	12	9.4%	
What are the symptoms that might prompt you to take your child to an eye specialist?					.501
Eye redness	91	26.6%	24	18.8%	
Staring eyes	34	9.9%	18	14.1%	
Double vision complaints	60	17.5%	19	14.8%	
Playing with toys from a close distance	53	15.5%	22	17.2%	
Frequent rubbing of the eye	61	17.8%	28	21.9%	
Tilt the head to one side	30	8.8%	11	8.6%	
Excessive tear production	13	3.8%	6	4.7%	
Do you accept the child wearing glasses?					.099
Yes	271	79.2%	110	85.9%	
No	71	20.8%	18	14.1%	
Would you allow your child to undergo eye surgery if necessary?					.139^$^
Yes	222	64.9%	100	78.1%	
No, the operation cost	30	8.8%	5	3.9%	
No, fear of the outcome	42	12.3%	11	8.6%	
No, it would cause more damage to the eyes	9	2.6%	2	1.6%	
No, I'm not sure	34	9.9%	8	6.3%	
No, cultural and social barriers	5	1.5%	2	1.6%	

## Discussion

In this self-reported cross-sectional study, we aimed to assess parents' awareness and attitude toward pediatric eye diseases. A previous study reported poor knowledge in 78.2% of parents [7], whereas another study reported poor knowledge among 91.9% of parents [8]. Consistently, our findings showed that 72.8% of parents had a low level of awareness regarding pediatric eye diseases. However, a similar study conducted in Saudi Arabia showed that 56.7% of the participated parents had an adequate understanding of children's eye diseases [11].

The results of the previous studies regarding this subject reveal the need for further assessment of the knowledge about children's eye diseases among parents and its associated factors to enhance the improvement of their understanding level [7,8,11].

According to our findings, parents aged 18-40 years showed a better knowledge level than older parents. In contrast, previous studies resulted in a higher level of knowledge among older parents in comparison to younger parents [1,7].

The parents' educational level was also associated with the level of knowledge since our study confirmed that participants below the university level had less awareness of pediatric eye diseases. Moreover, there was an apparent association between the respondents' employment status and their knowledge level, whereas employed parents showed more understanding of eye diseases among children. Furthermore, the association of higher family income with better awareness levels was also confirmed by another study [7].

A recent study indicates that the prevalence of refractive errors in Saudi Arabia is 17.5% affecting approximately one of every five children [14]. Furthermore, based on our results, 25.7% of the parents stated that their children have refractive errors. Accordingly, awareness of the causes, prevention measures, and management types of pediatric eye diseases should be raised.

Additionally, a significantly better understanding of pediatric eye diseases has been found among parents who have children with a history of eye disease [7]. This could be related to their continued contact with medical physicians as part of their management.

Sources of information that were used mainly by our study participants regarding pediatric eye diseases are physicians, followed by the internet. This is consistent with the results of a similar study. However, other studies have reported that the primary sources of information were family and friends [8,15].

The present study's findings showed that 81.1% of the parents accept their children wearing glasses. These findings are consistent with previously published articles [7,11]. However, dissimilarly, another study found that among the total number of parents who participated in their study, only 42.3% accepted the idea of wearing glasses [16].

Parents with good to excellent levels of knowledge showed more acceptance of their children wearing glasses than those with a lower level of knowledge. In addition, they stated their acceptance of their children undergoing eye surgery, if necessary. This fact confirms the importance of raising parents' awareness to manage pediatric eye diseases efficiently.

Limitations

A few limitations can be detected in this study; the difference between the participants' categories (gender and age) should be minimized to compare their results accurately, and the prevalence of common eye diseases should be assessed according to a more accurate method, such as medical records rather than self-reported data.

## Conclusions

Our study findings show inadequate parents’ knowledge about common pediatric eye diseases and their practice regarding eye care in children in Makkah, Saudi Arabia. Parents are the first caregiver and first teachers of their children. Also, the child acquires health habits from their parents. Therefore, awareness programs and educational sessions targeting both parents in social media platforms and public places are recommended to raise awareness about eye diseases and attitudes toward seeking medical advice.
